# Quantum scaling for the metal–insulator transition in a two-dimensional electron system

**DOI:** 10.1038/s41598-024-63221-6

**Published:** 2024-06-01

**Authors:** V. Kagalovsky, S. V. Kravchenko, D. Nemirovsky

**Affiliations:** 1 Shamoon College of Engineering, 84105 Beer-Sheva, Israel; 2 Department of Physics, Northeastern University, Boston, MA 02115 USA

**Keywords:** Condensed-matter physics, Phase transitions and critical phenomena

## Abstract

The quantum phase transition observed experimentally in two-dimensional (2D) electron systems has been a subject of theoretical and experimental studies for almost 30 years. We suggest Gaussian approximation to the mean-field theory of the second-order phase transition to explain the experimental data. Our approach explains self-consistently the universal value of the critical exponent 3/2 (found after scaling measured resistivities on both sides of the transition as a function of temperature) as the result of the divergence of the correlation length when the electron density approaches the critical value. We also provide numerical evidence for the stretched exponential temperature dependence of the metallic phase’s resistivities in a wide range of temperatures and show that it leads to correct qualitative results. Finally, we interpret the phase diagram on the density-temperature plane exhibiting the quantum critical point, quantum critical trajectory and two crossover lines. Our research presents a theoretical description of the seminal experimental results.

## Introduction

In recent decades, quantum phase transitions have been among the most popular topics in condensed matter physics. They take place at zero temperature and are driven by the variation of the system’s physical parameters, such as pressure^1^, chemical doping^2,3^, or magnetic field^4^. Field theories for the quantum phase transitions are natural generalisations of Landau general theory of continuous second-order phase transitions^5^. They are presented in numerous books^6,7^, reviews^8^, and papers (see, e.g., Ref. ^9^ and references therein).

The quantum phase transition we consider in this paper is the zero-magnetic-field metal–insulator transition driven by variations in electron density, $n_{\text{ s }}$. It was first observed in a 2D electron system in high-mobility silicon metal-oxide-semiconductor field effect transistors (MOSFETs)^10–12^ and subsequently in a wide variety of 2D electron and hole systems: *p*-type SiGe heterostructures, *p*- and *n*-type GaAs/AlGaAs heterostructures, AlAs heterostructures, ZnO-related heterostructures, etc. (for reviews, see, e.g., Refs. ^13–20^). The most substantial drop of the resistance on the metallic side of the transition (up to a factor of 12) at sub-Kelvin temperatures was reported in 2D systems in ultra-clean SiGe/Si/SiGe quantum wells^21^. The first attempt to scale the experimental data, made in Refs. ^11,12,22^, suggested that the metal–insulator transition is a genuine quantum phase transition. Strictly speaking, near the critical density, on the metallic side of the transition, the *R*(*T*) curves are non-monotonic and exhibit a maximum at a specific temperature $T_{\text {max}}$. Here, we focus only on data obtained at temperatures below $T_{\text {max}}$; the scaling analysis of the entire *R*(*T*) curves on the metallic side of the transition was made in Refs. ^23,24^ in the spirit of the renormalization-group theory^19,25,26^ and the dynamical mean-field theory^18,27–29^.

## Fitting the experimental resistivity data

Quantum phase transition driven by the electron density $n_s$ manifests itself by the fact that the resistivity of a sample with critical density $n_c$ (at the metal–insulator transition) is temperature-independent. It implies that the resistivities of different samples as a function of temperature *T* can be presented as a unique function of a product of *T* and a function of $n_s$, which vanishes at $n_c$. As it was remarkably mentioned in Ref.^30^, the existence of this function is “a hallmark of quantum criticality.”

**Figure 1:**
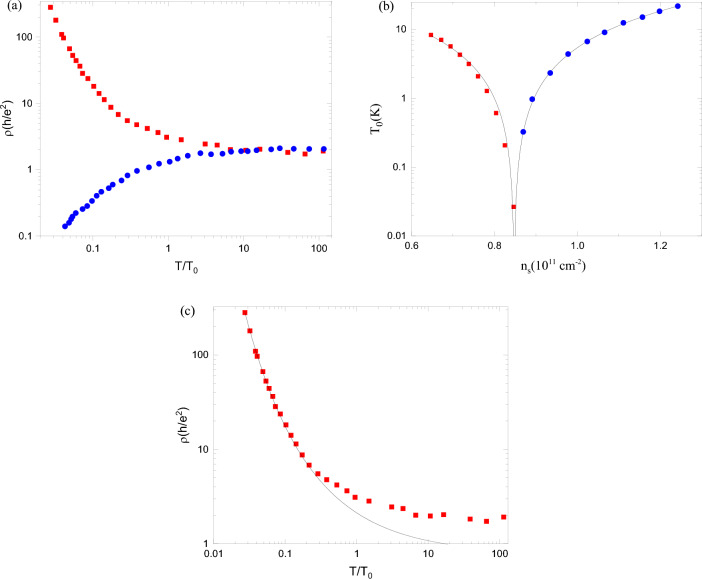
(**a**) Resistivities $\rho$ as functions of a rescaled temperature $T/T_0$. Full red squares are experimental data of the insulating phase ($n_{\text{ s }}<n_{\text{ c }}$), and full blue circles are experimental data of the metallic phase ($n_{\text{ s }}>n_{\text{ c }}$). (**b**) Scaling parameter $T_0$ as a function of electron density. Full red squares correspond to the insulating phase ($n_{\text{ s }}<n_{\text{ c }}$) and full blue circles to the metallic phase ($n_{\text{ s }}>n_{\text{ c }}$). (**c**) Resistivity $\rho$ of the insulating phase as a function of a rescaled temperature $T/T_0$. Full red squares are experimental data for $n_{\text{ s }}<n_{\text{ c }}$^12^. The black solid line is a numerical fit by the function $\rho _0\exp {((T_0/T)^\alpha )}$. The best fit for low temperatures is found for $\alpha \approx 1/2$.

**Figure 2:**
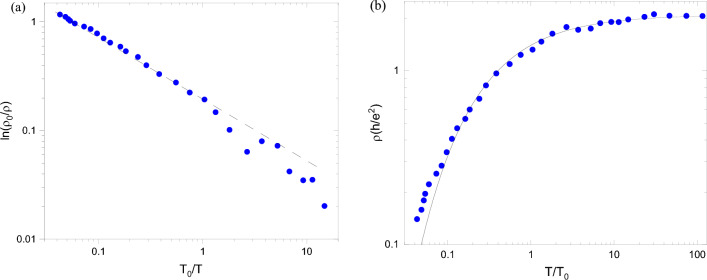
(**a**) Full blue circles are experimental data of the logarithm of inversed renormalised resistivity $\log (\rho _0/\rho )$ in the metallic phase ($n_{\text{ s }}>n_{\text{ c }}$) as a function of an inverse rescaled temperature $T_0/T$. The dashed straight line is a guideline for the eye. (**b**) Resistivity $\rho$ on the metallic phase as a function of a rescaled temperature $T/T_0$. Full blue circles are experimental data for $n_{\text{ s }}>n_{\text{ c }}$^12^. The black solid line is a numerical fit by a function $\rho _0\exp {(-\beta (T_0/T)^\gamma )}$. The best fit is found for $\gamma \approx 2/3$.

We have analysed the collapse (after proper scaling of the temperature) of all resistivity data on two curves (insulating and metallic) presented in Fig. 3 of Ref. ^12^. The resistivity (Fig. 1a) becomes a function of the rescaled temperature $T/T_0$ where the parameter $T_0$ (Fig. 1b) depends only on the electron density of the system. Following the original papers^11,12^, we fit data points for both resistivity regimes and the parameter $T_0$ with the functions described and explained below using both Levenberg-Marquardt and Trust-Region Reflective algorithms implemented in Matlab Curve Fitting Toolbox^31^ to cross-check the output fitting parameters. We fitted two sets of points for the dependences $T_0(n_{\text{ s }})$ with a single function $A|n_{\text{ s }}-n_{\text{ c }}|^{\nu _1}$ (here $n_{\text{ c }}$ is the critical density at which the metal–insulator transition occurs, measured in the experiment^12^) and provided the exponent $\nu _1=3/2$ almost precisely for both algorithms mentioned above. The resistivity in the insulating phase is characterised by the variable range hopping in the strongly interacting system^32^. To check whether hopping in the presence of a Coulomb gap^33^ defines the resistivity, we used a function

1 $$\begin{aligned} \rho =\rho _0\exp {(T_0/T)^\alpha } \end{aligned}$$

with two parameters, $\rho _0$ and $\alpha$. As expected, our fitting procedure indeed provided the power in the exponent $\alpha =1/2$ with great accuracy for the low-temperature data (see Fig. 1c). The fit of the resistivity in the metallic regime does not have such a rigorous theoretical background as hopping in the presence of a Coulomb gap^33^ in the insulator (mentioned above and discussed in detail below). To find a monotonic curve to fit experimental data, we have included only resistivities at temperatures that are not extremely low, $T/T_0>0.05$. The qualitative similarity (after reflection from the horizontal line $\rho =\rho _0$) of both curves in Fig. 1a led us to suggest that a stretched exponential function could also describe the metallic data. To check this suggestion, we redraw the data of the lower graph: the dependence of $\log (\rho _0/\rho )$ on $T_0/T$ is presented as a log–log plot in Fig. 2a. One can immediately observe that the data points are very close to a straight line, confirming our suggestion about a stretched exponential function. The resistivity data in the metallic phase are then fitted by the function

2 $$\begin{aligned} \rho =\rho _0\exp {(-\beta (T_0/T)^\gamma )} , \end{aligned}$$

containing three parameters. The fit is presented in Fig. 2b, and the value of the exponent $\gamma$ is very close to 2/3. We underline that this stretched exponential dependence is valid for temperatures $T/T_0>0.05$ and cannot describe the resistivity at very low temperatures. On the other hand, according to the renormalization-group theory^25,26^, the resistivity continues to decrease as the temperature approaches zero. It is also important to cite that the stretched exponential scaling (Eq. (2)) has been studied for the metal–insulator transition in the Hubbard model^30,34,35^ and in the 2D kappa organic systems^36^. It was found that the scaling holds on both sides of the transition, similar to our results.

## The model: order parameter, critical exponents, and phase diagram

Landau theory of the second-order phase transitions^5^ defines the order parameter $\phi$ to characterise the phase with broken symmetry. The most prominent examples of the order parameter are magnetisation in ferromagnetic phase transition and pseudowavefunction in metal–superconductor phase transition (the square of its absolute value is a measure of the local density of superconducting electrons). In the first step, the theory neglects fluctuations and introduces free energy density expanded in even powers of the order parameter

3 $$\begin{aligned} F=a\phi ^2+b\phi ^4. \end{aligned}$$

The central assumption of the theory is that the factor *a* multiplied by the square of the order parameter in the free energy functional^5,7,37^ changes its sign when the temperature crosses a critical value $T_c$ (linear dependence $a=a_0(T-T_c)$) and that the next term is the product of the fourth power of the order parameter and a constant positive prefactor *b*. This dependence leads to the minimum free energy in a symmetry-broken phase (ferromagnet and superconductor in the above examples) to the order parameter $\phi _0$ being proportional to the square root of the difference between critical and actual temperatures

4 $$\begin{aligned} \phi _0=\sqrt{-\frac{a}{2b}}=\sqrt{\frac{a_0}{2b}(T_c-T)} \end{aligned}$$

For quantum transitions, the effective action replaces the free energy functional of Landau theory^7^, and the distance to the critical point plays the role of the temperature difference. The quantum phase transition we describe in this Letter is a metal–insulator transition driven by the change in electron density. The definition of the order parameter for a metal–insulator transition is not as straightforward as for a typical second-order phase transition. Effectively, the order parameter must be related to the number of free charge carriers. The average of the order parameter has to be finite in the metallic phase, vanishes when the electron density approaches its critical value and remains zero in the insulating phase. The derivation of the correlation length of the order parameter presented below explains the value of the critical exponent 3/2 derived from the experimental data. To define the order parameter, we follow the typical medium theory^38,39^. That theory is based on the divergence of the typical escape rate when the metal–insulator transition is approached from the metallic side, as first noted in the seminal paper by Anderson^40^. The order parameter is a typical local density of states^38^ (inversely proportional to the typical escape rate), determining the conductivity. This definition of the order parameter is in the spirit of Landau theory: non-zero order parameter in a metallic phase and order parameter equal to zero in the insulating phase. Numerical studies showed the equality of critical exponents describing the behaviour of the physical parameters when the system approaches quantum phase transition: the order parameter and conductivity tend to zero, and correlation length diverges on the metallic side of the transition and of the localisation length diverges on the insulating side^38,39^. The value of the critical exponent $\nu _1=3/2$ found from the fitting of the experimental data leads to the assumption that the effective distance to the critical point is proportional to the cube of the difference between critical and actual electron densities, $(n_{\text{ c }}-n_{\text{ s }})^3$. As shown below, the effective action with this definition of the effective distance explains the value of the critical exponent self-consistently. Gaussian approximation considers fluctuations of the order parameter neglected in the free energy functional. Its application to the quantum phase transition leads to the following expression for the action density

5 $$\begin{aligned} F=a\phi ^2+b\phi ^4+c(\nabla \phi )^2, \end{aligned}$$

where $a=a_0(n_{\text{ c }}-n_{\text{ s }})^3$, and the parameters $a_0$, *b*, and *c* are positive constants. First, we consider the action density without fluctuations. Minimisation of the first two terms in Eq. (5) (in the spirit of the Landau theory) immediately provides zero averaged order parameter $\phi _0=0$ in the insulating phase ($n_{\text{ s }}<n_{\text{ c }}$) and finite value in the metallic phase ($n_{\text{ s }}>n_{\text{ c }}$)

6 $$\begin{aligned} \phi _0=\sqrt{-\frac{a}{2b}}. \end{aligned}$$

The addition of the square of the gradient of the order parameter in the Gaussian approximation to the second-order phase transition allows one to find the correlation length $\xi _{\text {cor}}$ of the order parameter^41^. The minimum of the functional in Eq. (5) corresponds to the Euler variational equation

7 $$\begin{aligned} -c\Delta \phi +a\phi +b\phi ^3=0. \end{aligned}$$

Small deviations $\phi _1(\textbf{x})$ of the order parameter from its average value $\phi _0$ satisfy the following equation

8 $$\begin{aligned} -c\Delta \phi _1+(a+3b\phi _0^2)\phi _1=0. \end{aligned}$$

In the presence of the point source, the solution of Eq. (8) is a Green’s function. It is proportional to the correlation function of the order parameter $\langle \phi (\textbf{r})\phi (0)\rangle$. For a two-dimensional system, it leads to a modified Bessel function of the second kind of zero-order

9 $$\begin{aligned} \langle \phi (\textbf{r})\phi (0)\rangle \sim K_0\left( r/\xi _{\text {cor}}\right) . \end{aligned}$$

An asymptotic expansion of function $K_0$ at large distances *r* leads to the exponential decay of the correlation function

10 $$\begin{aligned} \langle \phi (\textbf{r})\phi (0)\rangle \sim \sqrt{\frac{\pi \xi _{\text {cor}}}{2r}}\exp \left( -r/\xi _{\text {cor}}\right) , \end{aligned}$$

confirming that the parameter $\xi _{\text {cor}}$ is indeed the correlation length. It follows from Eq. (8)

11 $$\begin{aligned} \xi _{\text {cor}}=(c/(a+3b\phi _0^2))^{1/2}. \end{aligned}$$

The dependence of the correlation length on the concentration difference is identical (the same divergence exponent $\nu _2=3/2$) on both sides of the transition (the amplitude on the insulating side is twice larger than on the metallic one)

12 $$\begin{aligned} \xi _{\text {cor}}\sim |n_{\text{ s }}-n_{\text{ c }}|^{-3/2} \end{aligned}$$

This value of the critical exponent, $\nu _2$, equals the exponent $\nu _1=3/2$ for the scaling parameter $T_0$ found from the experimental data on both sides of the transition, as discussed above. This equality is the most natural result for the insulating phase: indeed, in the variable range hopping regime, the scaling parameter $T_0$ is inversely proportional to the localisation length, according to Ref. ^33–36^ and the correlation length we find is similar to the localisation length for the insulator. We should also mention that various numerical simulations of the metal–insulator transition in three dimensions find the critical exponent values close to 3/2^38,39,42–45^ and also provide equal critical exponents on both sides of the transition.

We wish to underline that our choice of parameters in the effective action (Eq. 5) is not unique. One can choose two or all three coefficients to be dependent on the electron density to produce a critical exponent 3/2 in Eq. (11). Our assumption of the effective distance to the critical point to be proportional to the cube of the difference between critical and actual electron densities (coefficient *a*) seems like the simplest one while keeping two other parameters (*b* and *c*) constant is in the spirit of Landau theory.

Now, we consider the temperature dependence of the resistivity of the metallic phase. We have to underline that the exponential function in Eq. (2) is suggested as a fit for the first time and show below that the numerically found value of the power in the exponent $\gamma \approx 2/3$ leads to the correct physical results. In the vicinity of the critical density, the difference in the conductivities $\sigma =1/\rho$ can be approximated as

13 $$\begin{aligned} \sigma -\sigma _0=\frac{1}{\rho _0}(\exp (\beta (T_0/T)^{\gamma })-1)\approx \frac{\beta }{\rho _0}\left( \frac{T_0}{T}\right) ^{\gamma }\sim T_0^{\gamma }\sim (n_{\text{ s }}-n_{\text{ c }})^{\gamma \nu _1}\approx (n_{\text{ s }}-n_{\text{ c }}). \end{aligned}$$

The fact that the difference in conductivities is linearly proportional to the difference in electron densities is a rigorous result for ordinary conductors with conductivity satisfying the Drude formula. Then, one can speculate that this linear difference remains approximately correct in the metallic phase of the interacting system close to the transition.

**Figure 3:**
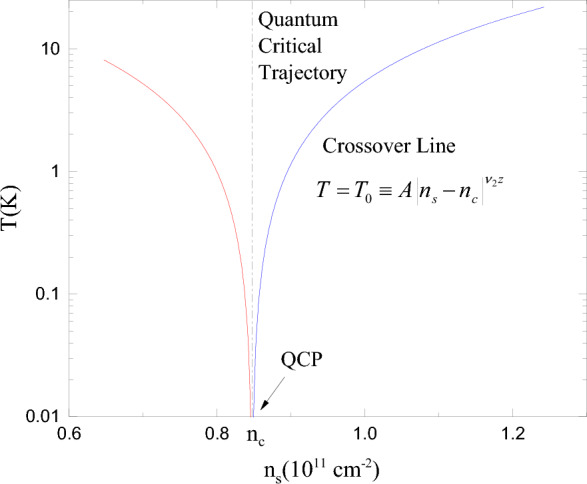
Phase diagram of the system. The quantum critical point (QCP) is on the horizontal axis at $n_{\text{ c }}\approx 0.85$, $T=0$. Quantum critical trajectory is a vertical black dash-dotted line starting at QCP. Crossover lines are represented by solid red (insulator) and blue (metal) lines.

Finally, we discuss the system phase diagram. To do so, we redraw Fig. 1b with the vertical axis as the temperature, mark the critical density $n_{\text{ c }}$ on the horizontal axis, and draw a vertical line $n_{\text{ s }}=n_{\text{ c }}$. Fig. 3 is a typical phase diagram of the system exhibiting a quantum phase transition^1,4,7^. It takes place at zero temperature when the density crosses the critical one. Vertical line $n_{\text{ s }}=n_{\text{ c }}$ is a quantum critical trajectory with temperature-independent resistivity. Two lines $T=T_0\equiv A|n_{\text{ s }}-n_{\text{ c }}|^{\nu _1}$ represent crossover lines in the non-critical region of the phase diagram. The crossover line is a characteristic feature of a quantum phase transition defined by the following expression: $T\sim |n_{\text{ s }}-n_{\text{ c }}|^{\nu _2z}$^1,7^, where *z* is a dynamic exponent. It was measured for a two-dimensional electron system^46^ and found to be very close to the theoretical value of the dynamical exponent in a strongly interacting two-dimensional system $z=1$^47^. In our phase diagram with $\nu _2=\nu _1=3/2$ and $z=1$, these lines separate distinct regions of the order parameter. Well below the line at $T\ll T_0$ on the insulating side, the localisation length has finite temperature-independent values along vertical lines. The localisation length is infinite below the line on the metallic side of the transition and does not depend on temperature or electron density. Above the crossover lines, the localisation length acquires temperature dependence.

## Conclusions

We have proposed the effective action density to describe the metal–insulator phase transition in a two-dimensional strongly interacting electron system. We have defined the effective distance to the quantum critical point as the cube of the difference between the critical and actual electron densities. Using this assumption, we found analytically the value of the critical exponent for the divergence of the correlation length, which coincides with the experimental data on both sides of the transition. This critical exponent describes the divergence of the localisation length in the variable range hopping regime observed in the experiment for the insulating phase. We have suggested for the first time that the stretched exponential function can also describe the resistivity dependence on the temperature in the metallic phase in a wide range of temperatures for the experimental data^12^. The numerically evaluated value of the power in the exponent correctly explains the resistivity dependence on the electron density. We have described a phase diagram in an electron density-temperature plane as a typical phase diagram of a system exhibiting a quantum phase transition. Concerning the application to other experiments, our approach shows that the critical exponent of the correlation length (if it can be inferred based on measurement) can identify the dependence of the effective distance to the critical point on the electron density that drives the transition.

## Methods

Data points for both metallic and insulating regimes and the parameter $T_0$ were fitted using advanced machinery of Matlab Curve Fitting Toolbox^31^.

## Data Availability

The data supporting this study’s findings are available from the corresponding author upon reasonable request.

## References

[1] Garst M, Rosch A (2005). Sign change of the Grüneisen parameter and magnetocaloric effect near quantum critical points. Phys. Rev. B.

[2] Freitas DC (2015). Experimental consequences of quantum critical points at high temperatures. Phys. Rev. B.

[3] Küchler R (2003). Divergence of the Grüneisen ratio at quantum critical points in heavy fermion metals. Phys. Rev. Lett..

[4] Zajarniuk T (2022). Quantum versus classical nature of the low-temperature magnetic phase transition in $${TbAl}_{3}{({\rm BO }_{3})}_{4}$$. Phys. Rev. B.

[5] Landau LD, Lifshitz EM (1980). Statistical Physics Part 2.

[6] Sachdev S (2011). Quantum Phase Transitions.

[7] Continentino M (2017). Quantum Scaling in Many-Body Systems: An Approach to Quantum Phase Transitions.

[8] Senthil, T. Deconfined quantum critical points: A review (2023). arXiv cond-mat 2306.12638.

[9] Sachdev S, Read N, Oppermann R (1995). Quantum field theory of metallic spin glasses. Phys. Rev. B.

[10] Zavaritskaya TN, Zavaritskaya ÉI (1987). Metal-insulator transition in inversion channels of silicon MOS structures. JETP Lett..

[11] Kravchenko SV, Kravchenko GV, Furneaux JE, Pudalov VM, D’Iorio M (1994). Possible metal-insulator transition at $$B=0$$ in two dimensions. Phys. Rev. B.

[12] Kravchenko SV (1995). Scaling of an anomalous metal-insulator transition in a two-dimensional system in silicon at $$B = 0$$. Phys. Rev. B.

[13] Abrahams E, Kravchenko SV, Sarachik MP (2001). Metallic behavior and related phenomena in two dimensions. Rev. Mod. Phys..

[14] Kravchenko SV, Sarachik MP (2004). Metal-insulator transition in two-dimensional electron systems. Rep. Prog. Phys..

[15] Spivak B, Kravchenko SV, Kivelson SA, Gao XPA (2010). Transport in strongly correlated two dimensional electron fluids. Rev. Mod. Phys..

[16] Shashkin AA, Kravchenko SV (2019). Recent developments in the field of the metal-insulator transition in two dimensions. Appl. Sci..

[17] Shashkin AA, Kravchenko SV (2021). Metal-insulator transition and low-density phases in a strongly-interacting two-dimensional electron system. Ann. Phys..

[18] Tan Y, Dobrosavljevi V, Rademaker L (2022). How to recognize the universal aspects of Mott criticality?. Crystals..

[19] Finkel’stein AM, Schwiete G (2023). Scale-dependent theory of the disordered electron liquid. Ann. Phys..

[20] Pradhan NR (2023). Insulator-to-metal phase transition in a few-layered MoSe_2_ field effect transistor. Nanoscale.

[21] Melnikov MY (2019). Quantum phase transition in ultrahigh mobility SiGe/Si/SiGe two-dimensional electron system. Phys. Rev. B.

[22] Popović D, Fowler AB, Washburn S (1997). Metal-insulator transition in two dimensions: Effects of disorder and magnetic field. Phys. Rev. Lett..

[23] Anissimova S, Kravchenko SV, Punnoose A, Finkel’stein AM, Klapwijk TM (2007). Flow diagram of the metal-insulator transition in two dimensions. Nat. Phys..

[24] Shashkin AA (2020). Manifestation of strong correlations in transport in ultraclean SiGe/Si/SiGe quantum wells. Phys. Rev. B.

[25] Punnoose A, Finkel’stein AM (2001). Dilute electron gas near the metal-insulator transition: Role of valleys in silicon inversion layers. Phys. Rev. Lett..

[26] Punnoose A, Finkel’stein AM (2005). Metal-insulator transition in disordered two-dimensional electron systems. Science.

[27] Camjayi A, Haule K, Dobrosavljević V, Kotliar G (2008). Coulomb correlations and the Wigner-Mott transition. Nat. Phys..

[28] Radonjić MM, Tanasković D, Dobrosavljević V, Haule K, Kotliar G (2012). Wigner-Mott scaling of transport near the two-dimensional metal-insulator transition. Phys. Rev. B.

[29] Dobrosavljević, V. & Tanasković, D. Wigner-Mott quantum criticality: From 2D-MIT to ^3^He and Mott organics. In *Kravchenko, S. V.* (ed.) *Strongly Correlated Electrons in Two Dimensions*, chap. *1*, 1–46 (*Pan Stanford Publishing*, 2017).

[30] Vučičević J, Terletska H, Tanasković D, Dobrosavljević V (2013). Finite-temperature crossover and the quantum Widom line near the Mott transition. Phys. Rev. B.

[31] MathWorks. *Curve Fitting Toolbox* (*Natick, Massachusetts, United States*, 2020).

[32] Mason W, Kravchenko SV, Bowker GE, Furneaux JE (1995). Experimental evidence for a Coulomb gap in two dimensions. Phys. Rev. B.

[33] Efros AL, Shklovskii BI (1975). Coulomb gap and low temperature conductivity of disordered systems. J. Phys. C Solid State Phys..

[34] Terletska H, Vučičević J, Tanasković D, Dobrosavljević V (2011). Quantum critical transport near the Mott transition. Phys. Rev. Lett..

[35] Vučičević J, Tanasković D, Rozenberg MJ, Dobrosavljević V (2015). Bad-metal behavior reveals Mott quantum criticality in doped Hubbard models. Phys. Rev. Lett..

[36] Furukawa T, Miyagawa K, Taniguchi H, Kato R, Kanoda K (2015). Quantum criticality of Mott transition in organic materials. Nat. Phys..

[37] Wilson KG (1975). The renormalization group: Critical phenomena and the Kondo problem. Rev. Mod. Phys..

[38] Dobrosavljević V, Pastor AA, Nikolić BK (2003). Typical medium theory of Anderson localization: A local order parameter approach to strong-disorder effects. Europhys. Lett..

[39] Janssen M (1998). Statistics and scaling in disordered mesoscopic electron systems. Phys. Rep..

[40] Anderson PW (1958). Absence of diffusion in certain random lattices. Phys. Rev..

[41] Patashinskij AZ, Pokrovsky VL (1979). Fluctuation Theory of Phase Transitions.

[42] MacKinnon A (1994). Critical exponents for the metal-insulator transition. J. Phys. Condens. Matter..

[43] Slevin K, Ohtsuki T (2014). Critical exponent for the Anderson transition in the three-dimensional orthogonal universality class. New J. Phys..

[44] Rodriguez A, Vasquez LJ, Slevin K, Römer RA (2010). Critical parameters from a generalized multifractal analysis at the Anderson transition. Phys. Rev. Lett..

[45] Rodriguez A, Vasquez LJ, Slevin K, Römer RA (2011). Multifractal finite-size scaling and universality at the Anderson transition. Phys. Rev. B.

[46] Kravchenko SV, Simonian D, Sarachik MP, Mason W, Furneaux JE (1996). Electric field scaling at a $$B=0$$ metal-insulator transition in two dimensions. Phys. Rev. Lett..

[47] Sondhi SL, Girvin SM, Carini JP, Shahar D (1997). Continuous quantum phase transitions. Rev. Mod. Phys..

